# Role of TFRC as a Novel Prognostic Biomarker and in Immunotherapy for Pancreatic Carcinoma

**DOI:** 10.3389/fmolb.2022.756895

**Published:** 2022-03-16

**Authors:** Cheng Yang, Junqiang Li, Yongdong Guo, Dongxue Gan, Chao Zhang, Ronglin Wang, Lei Hua, Liaoliao Zhu, Peixiang Ma, Jingjie Shi, Shanshan Li, Haichuan Su

**Affiliations:** Department of Oncology, Tangdu Hospital, Air Force Medical University, Xi’an, China

**Keywords:** TFRC, pancreatic cancer, bioinformatics, immunotherapy, CAR- T cells

## Abstract

**Objective:** To explore the expression of the transferrin receptor (*TFRC*) gene in pancreatic cancer and to analyze the pathogenesis and immunotherapy of TFRC in patients using bioinformatics methods.

**Methods:** We used public data from the cancer genome atlas (TCGA) and gene expression omnibus databases to explore the expression level of the *TFRC* gene in pancreatic cancer patients. At the same time, we analyzed the correlation between the *TFRC* gene expression and patient survival, and further analyzed the correlation between *TFRC* and survival time of patients with different clinicopathological characteristics. Co-expressed genes and pathway enrichment analyses were used to analyze the mechanism of the TFRC in the occurrence and development of pancreatic cancer. Ultimately, we used the R software to examine the relationship between TFRC and immune phenotypes and immune cell infiltration using the TCGA database.

**Results:** The results of the study showed that TFRC is highly expressed in pancreatic cancer tissue. The upregulated expression of TFRC was negatively correlated with the survival in patients with pancreatic cancer. The bioinformatics analysis showed that TFRC plays a role in the occurrence and development of pancreatic cancer mainly through signaling pathways (including cell adhesion molecule binding, condensed chromosomes, chromosome segregation, and cell cycle checkpoints). Finally, TFRC is associated with immune phenotypes and immune cell infiltration, which may influence immunotherapy.

**Conclusion:** TFRC is significantly increased in pancreatic cancer and is associated with a poor prognosis. Moreover, research on TFRC may generate new ideas for the immunotherapy of pancreatic cancer.

## Background

As a common gastrointestinal malignancy, pancreatic cancer is the 10th most common malignancy in the US. (Siegel et al., 2021) resulting in a high mortality rate over the past 20 years (Mizrahi et al., 2020). The occurrence and development of pancreatic cancer is a complex process with multi-stage and multi-gene involvement caused by a variety of factors, including the comprehensive effect of various external factors such as inflammation and diet, as well as internal factors such as gene mutations (Chen et al., 2020; Huber et al., 2020; Mizrahi et al., 2020). Although chemotherapy, radiotherapy, and immunotherapy (Schizas et al., 2020) for pancreatic cancer (Heinrich and Lang, 2017; Hosein et al., 2020) were broadly studied in the recent years, the early symptoms of pancreatic cancer are not clear and there is a lack of knowledge about specific biomarkers (Lee & Lee., 2014; Pereira et al., 2020; Zhang et al., 2018). Due to this, patients are often diagnosed at an advanced stage (Ansari et al., 2016; Chu et al., 2017; Goral, 2015; Wolfgang et al., 2013) with a poor prognosis (Mizrahi et al., 2020). Thus, specific biomarkers for the diagnosis and prognosis of pancreatic cancer should be urgently explored.

The transferrin receptor (*TFRC*) gene encodes a cell surface receptor that is required for cellular iron uptake. The receptor transports iron from the outside to the inside of the cell via the receptor-mediated endocytosis, which is important for cell growth. At the same time, TFRC has multiple alternatively spliced variants. TFR1 expressed highly in erythroblasts and rapidly proliferating cells, such as cancer cell. As a type II transmembrane glycoprotein, it can mediate endocytosis of iron bound by transferrin (TF), playing a major role in iron uptake (Kawabata, 2019). Conversely, although TFR2 is expressed in a variety of tissues, it plays a lesser role in iron transport. A previous study used quantitative seroproteomics to identify antibody biomarkers in pancreatic tissue and found that TFRC is a pancreatic cancer-associated antigen (Jhaveri et al., 2016). Another study reported that (Camp et al., 2013) transferrin receptors targeting wild-type p53 gene nanomedicines make pancreatic cancer sensitive to gemcitabine therapy. Moreover, TFRC can be used as a malignant marker of pancreatic cancer and pancreatic neuroendocrine tumor (Ryschich et al., 2004). However, the mechanism of TFRC in pancreatic cancer and its relationship with clinicopathological parameters in pancreatic cancer remain unclear.

As a new type of cancer therapy, immunotherapy has achieved good results in various cancers (Riley et al., 2019). However, because of the unique tumor microenvironment and low cancer immunogenicity in pancreatic cancer (Wang et al., 2020), single-agent immunotherapy is not effective in treating pancreatic cancer (Bear et al., 2020). Novel immunotherapy strategies include immune checkpoint inhibitors, cancer vaccines, adoptive cell transfer, combinations with other immunotherapeutic agents, chemoradiotherapy, or other molecularly targeted agents (Schizas et al., 2020). We think TFRC may play a role in various immunotherapy as a membrane protein associated with iron death.

This study deployed large sample data from databases and biochemical experiments to explore the expression level and clinical application of TFRC in pancreatic cancer. We also analyzed the mechanism of TFRC and its role in immunotherapy using bioinformatics technology to provide suggestions for pancreatic cancer.

## Methods

### Patient Samples

We obtained three pairs of surgically resected pancreatic cancers and corresponding peritumoral tissues from the Xi-jing Hospital and immediately stored them in liquid nitrogen. The patients that met the 2020 cancer diagnostic criteria signed informed consent before the experiment. The experiment was approved by the ethics committee of the Air Force Military Medical University.

### Pancreatic Cell Line Culture

Normal pancreatic cells (HPDE6-C7) and pancreatic cancer cell lines (PANC-1, MIAPaCa-2, PaTu8988T, SW 1990, Canpan-2, BXPC-3, HPAF-II, and Capan-1) were obtained from Procell Life Science and Technology Company (Wuhan, China) and BeNa Culture Collection (BNCC, Beijing, China). The cells were cultured in the recommended medium (10% fetal bovine serum) and kept in a CO_2_-free humidifying incubator at 37°C.

### Western Blot Analysis

Normal pancreatic cells and pancreatic cancer cell lines were lysed using lysis buffer (Applygen, Beijing, China) with a phosphatase and protease inhibitor cocktail (Roche, Branchburg, USA). Sodium dodecyl sulphate–polyacrylamide gel electrophoresis was used to fractionate the protein samples, and the protein bands were transferred onto a gel. After being blocked by using 5% nonfat milk for 3 h, polyvinylidene difluoride (PVDF) membranes were incubated using antibodies TFRC (1:1,000, 10084-2-AP, proteintech) and glyceraldehyde 3-phosphate dehydrogenase (1:1,0000, AC002, abcolone) overnight at 4°C. Then, we used horseradish peroxidase-conjugated secondary antibodies to incubate the PVDF membranes. In the end, the protein bands were detected using a Tanon-5200 hemiluminescent system (Shanghai, China).

### Immunohistochemistry

Tumor tissue sections and peritumoral tissue sections were deparaffinized using xylene and then rehydrated using different concentrations of ethanol. We inhibited endogenous hydrogen peroxide activity with 3% hydrogen peroxide and immediately repaired the antigen using hot citric acid buffer. Then, 5% normal goat serum was used to block the tissue sections. The sections were subsequently washed in phosphate-buffered saline (PBS) and incubated overnight at 4°C with the antibody against TFRC (1:100, 10084-2-AP, Proteintech) and CD3 (1:6400, 60181-1-Ig, Proteintech). On the next day, after washing in PBS solution for 10 min, the peroxidase-conjugated secondary antibody was used to incubate the microarray and sections for 30 min. Then, these tissue microarray and tumor tissue sections were stained with diaminobenzidine for 3 min. Finally, the slides were counterstained with haematoxylin.

### Gene Expression and Clinical Data

We downloaded the mRNA sequencing data and associated clinical information from the cancer genome atlas (TCGA) database, which involved 177 tumor tissues and four normal tissues. Furthermore, we downloaded other mRNA expression data from the GSE15471, GSE16515, GSE62452 and GSE45757 datasets from the gene expression omnibus (GEO) database. As these data are public network resources, the use of this particular data did not require any ethical approval or informed consent.

### Survival Analysis of Pancreatic Cancer Patient

We used GEPIA (a web-based tool for analyzing the data provided by TCGA) to analyze the prognostic value of TFRC expression in pancreatic adenocarcinoma (PAAD) patients and calculate the hazard ratio (HR) with 95% confidence and log-rank *p*-value. The statistical package for the social sciences (SPSS) software was applied for multivariate and univariate analyses based on the TCGA data to determine whether it was an independent prognostic factor or not.

### Profiling of Genes and miRNA Co-expressed With TFRC

To further explore the TFRC-related molecular mechanisms in pancreatic cancer, we used the cBioPortal database (https://www.cbioportal.org/) to identify co-expressed genes and related miRNAs. Subsequently, we selected 10 genes and several miRNAs that were significantly correlated with TFRC for further analysis and verified their correlation with TFRC in the tumor immune estimation resource (TIMER) database.

### Establishment of a Protein Interaction Network

We used STRING (a search tool for the retrieval of interacting genes) to construct the TFRC and related gene–protein interaction network with several aspects (interaction, co-expression, and co-localization). After the standard network diagram was constructed, the Cytoscape software was imported to beautify the network diagram.

### Pathway Enrichment Analysis of TFRC

We used the David (https://david.ncifcrf.gov/) online database to analyze the gene ontology (GO) and kyoto encyclopedia of genes and genomes (KEGG) pathways of *TFRC* and related genes. GO analysis includes biological processes (BP), molecular functions (MF), and cellular components (CC). Statistical significance was set at *p* < 0.05, while data visualization was executed using the GraphPad Prism5 software.

### Evaluation the Role of TFRC in Immunotherapy of PAAD

We evaluated the immune characteristics of TFRC in pancreatic cancer from four aspects: infiltration level of tumor infiltrating immune cells (TIICs), expression of inhibitory immune checkpoints, expression of immunomodulators, and activity of the cancer immunity cycle. The invasion level of TIICs in the tumor microenvironment was calculated using a variety of algorithms based on bulk RNA-seq data. Then, we examined the relationship between TFRC and common immune checkpoints (CTLA4, LAG3, PD1, and PDL1) in pan-cancer. We also collected information on 144 immunomodulators, including immune inhibitors, immune stimulators, MHC, chemokines, and receptors, and analyzed their relationship. Previous studies (Hu et al., 2021) have reported that a cancer immunity cycle reflects the anticancer immune response and comprises seven steps: release of cancer cell antigens (Step 1), cancer antigen presentation (Step 2), priming and activation (Step 3), trafficking of immune cells to tumors (Step 4), infiltration of immune cells into tumors (Step 5), recognition of cancer cells by T cells (Step 6), and killing of cancer cells (Step 7). We analyzed the correlation between TFRC and the cancer immunity cycle. We further evaluated the role of TFRC in immunotherapy by selecting some immune-checkpoint-relevant and immune-activity-relevant genes (Zhang et al., 2020) in PAAD. In addition, TIDE (Jiang et al., 2018) is a computational method to model two primary mechanisms of tumor immune evasion, we analyzed the correlation between TFRC and Interferon-gamma (IFNG) signature, microsatellite instability (MSI) signature, macrophage M2 type and TIDE score.

### Statistical Analyses

We used SPSS 20.0, GraphPad Prism 5 and R version 4.1.0 software to perform the data analysis and create plots and figures. We used the mean ± SD (standard deviation) to express the measurement characteristics. This is the basis for the analysis of the differential expression of TFRC in tumors and non-tumors of pancreatic cancer patients from the TCGA and GEO databases by Student’s t test. Moreover, the association between TFRC and clinical characteristic variables was analyzed using the Pearson chi-squared test. At the last step, the univariate and multivariate analyses of TFRC were conducted using Cox proportional hazards analysis.

## Results

### TFRC Is Highly Expressed in PAAD

**FIGURE 1:**
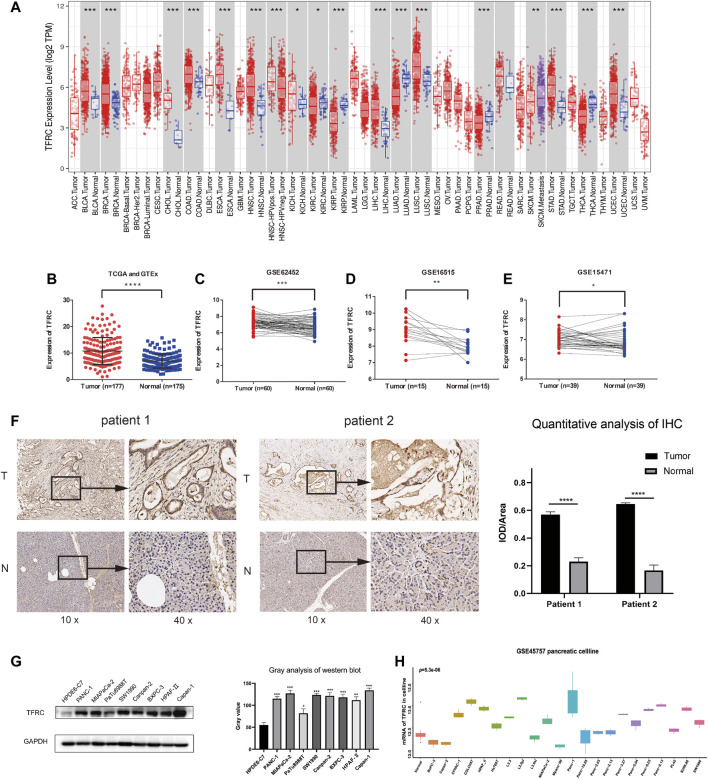
TFRC expression is elevated in pancreatic cancer. **(A)** TFRC mRNA is significantly higher in several cancer, including PAAD. **(B)** The difference in TFRC mRNA between pancreatic cancer and normal pancreas is significant (*p* < 0.0001). **(C–E)** In GEO database (GSE62452, GSE16515, GSE15471), TFRC mRNA is higher in pancreatic cancer tissues than corresponding peritumoral tissues (*p* = 0.0003, *p* = 0.0012, *p* = 0.0185). **(F)** Representative IHC staining of TFRC in matched PAAD and peritumoral tissues. **(G)** Western blotting shows TFRC protein expression levels in normal pancreatic cell (HPDE6-C7) and pancreatic cancer cell (PANC-1, MIAPaCa-2, PaTu8988T, SW 1990, Canpan-2, BXPC-3, HPAF-Ⅱ, Capan-1). **(H)** In GEO database (GSE45757), TFRC mRNA levels in normal pancreatic cell and pancreatic cancer cell.

We analyzed TCGA data related to TFRC expression levels in a variety of cancers (Figure 1A). Then, we investigated the difference in the expression levels of *TFRC* mRNA between pancreatic cancer and normal pancreas, and found that their levels were significantly higher in pancreatic cancer than that in normal pancreas (Figure 1B). Subsequently, we used the paired *t*-test to determine *TFRC* mRNA expression levels in pancreatic cancers and corresponding peritumoral tissues based on the data from the GEO database (Figures 1C–E). Then, we used immunohistochemistry to test the differences about TFRC protein levels in pancreatic tissues (Figure 1F). Besides, we found the same thing at the cellular level. The result of western blot showed that TFRC expression was significantly lower in normal pancreatic cell (HPDE6-C7) than that in pancreatic cancer cells (PANC-1, MIAPaCa-2, PaTu8988T, SW 1990, Canpan-2, BXPC-3, HPAF-II, Capan-1) (Figure 1G). Meanwhile, we verified *TFRC* mRNA levels in one pancreatic cell and 22 pancreatic cancer cell lines, which shows the *TFRC* mRNA levels were higher in most pancreatic cancer cell lines than in normal cell (Figure 1H).

### TFRC is a Diagnostic and Prognostic Indicator

**FIGURE 2:**
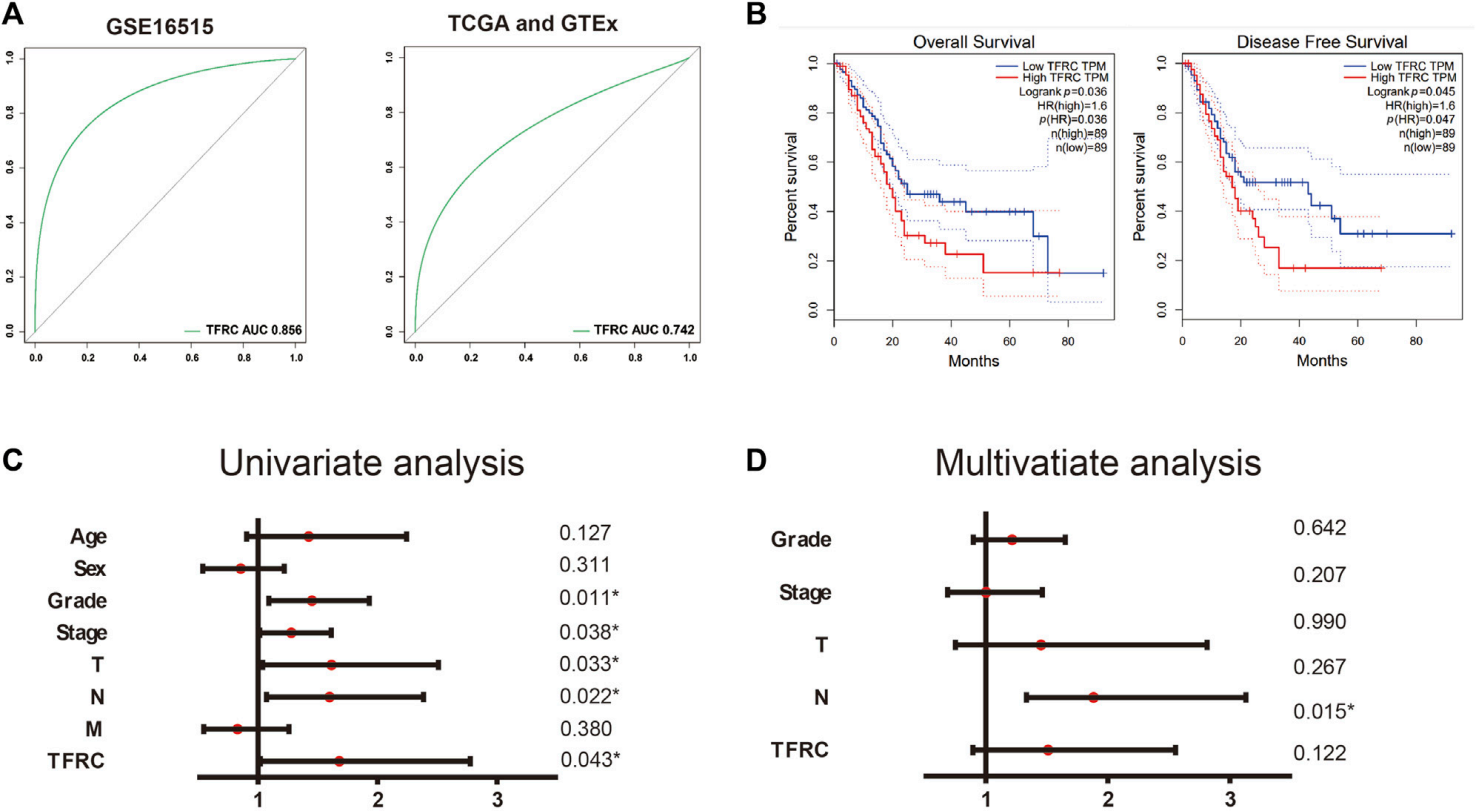
TFRC is a diagnostic and prognostic indicator. **(A)** ROC curve of TFRC about GSE16515 and TCGA. **(B)**. Kaplan-Meier overall survival analysis of TFRC expression in patients with PAAD using TCGA samples (n = 178, *p* = 0.036, log-rank test), Kaplan-Meier disease-free survival analysis of TFRC expression in patients with PAAD using TCGA samples (n = 178, *p* = 0.035, log-rank test). **(C, D)** Univariate (C) and multivariate (D) analyses of the relationship of TFRC expression and overall survival with clinicopathological characteristics in TCGA samples.

Firstly, we use TCGA and GEO database to build ROC curve, and found the AUC = 0.856 (GSE16515) and 0.742 (TCGA and GTEx), which means the TFRC has diagnostic significance (Figure 2A). Then, we used GEPIA and found that the patients with high TFRC expression had poor overall survival (*p* = 0.036, group cutoff = median) and poor disease-free survival (*p* = 0.045, group cutoff = median) (Figure 2B). At the same time, the univariate analysis showed that grade (HR = 1.451, *p* = 0.011), tumor node metastasis classification (TNM) stage (HR = 1.279, *p* = 0.038), invasion depth (T) stage (HR = 1.614, *p* = 0.033), and lymph node (N) stage (HR = 1.598, *p* = 0.022), and TFRC expression (HR = 1.681, *p* = 0.043) influenced the overall survival of patients. (Figure 2C). However, the multivariate analysis did not help in defining the upregulated TFRC expression as an independent prognostic factor (Figure 2D).

### Relationship Between TFRC Expression and Clinical Characteristics

To analyze the clinical value of TFRC in pancreatic cancer, we examined its relationship with the clinical factors of the patients. We used the median TFRC expression value to create a categorical dependent variable. The Pearson chi-squared test showed that TFRC expression was related to age (*p* = 0.001), pathological stage (Ⅰ vs. Ⅱ–Ⅳ, *p* = 5.97e-13), T stage (T1 vs. T2 vs. T3, T4, *p* = 0.047), and N stage (N0 vs. N1–Nx, *p* = 2.15e-05) (Table 1).

**TABLE 1:** Correlation of TFRC expression with clinicopathological features of PAAD.

Parameters	Total	TFRC expression		χ^2^	*p*-value
		LOW	HIGH		
Age					
≤60	58	28	30	10.5604	0.001155337^a^
>60	119	61	58		
Gender					
Female	80	36	44	0.8351	0.360815877
male	97	53	44		
Grade					
G1	30	19	11	4.3058	0.230277596
G2	95	49	46		
G3	48	19	29		
G4	2	1	1		
Stage					
Ⅰ	20	11	9	51.8571	5.97E-13^a^
Ⅱ-Ⅳ	153	75	78		
Invasion depth					
T1	7	5	2	6.0972	0.047425542^a^
T2	24	7	17		
T3, T4	144	77	67		
Lymph node metastasis					
N0	50	29	21	18.05	2.15E-05^a^
N1, Nx	126	59	67		
Distant metastasis					
M0	78	40	38	0.9529	0.328971774
M1, Mx	97	47	50		

a : p < 0.05, statistically significant.

### Analysis of Genes Co-Expressed With TFRC in PAAD

**FIGURE 3:**
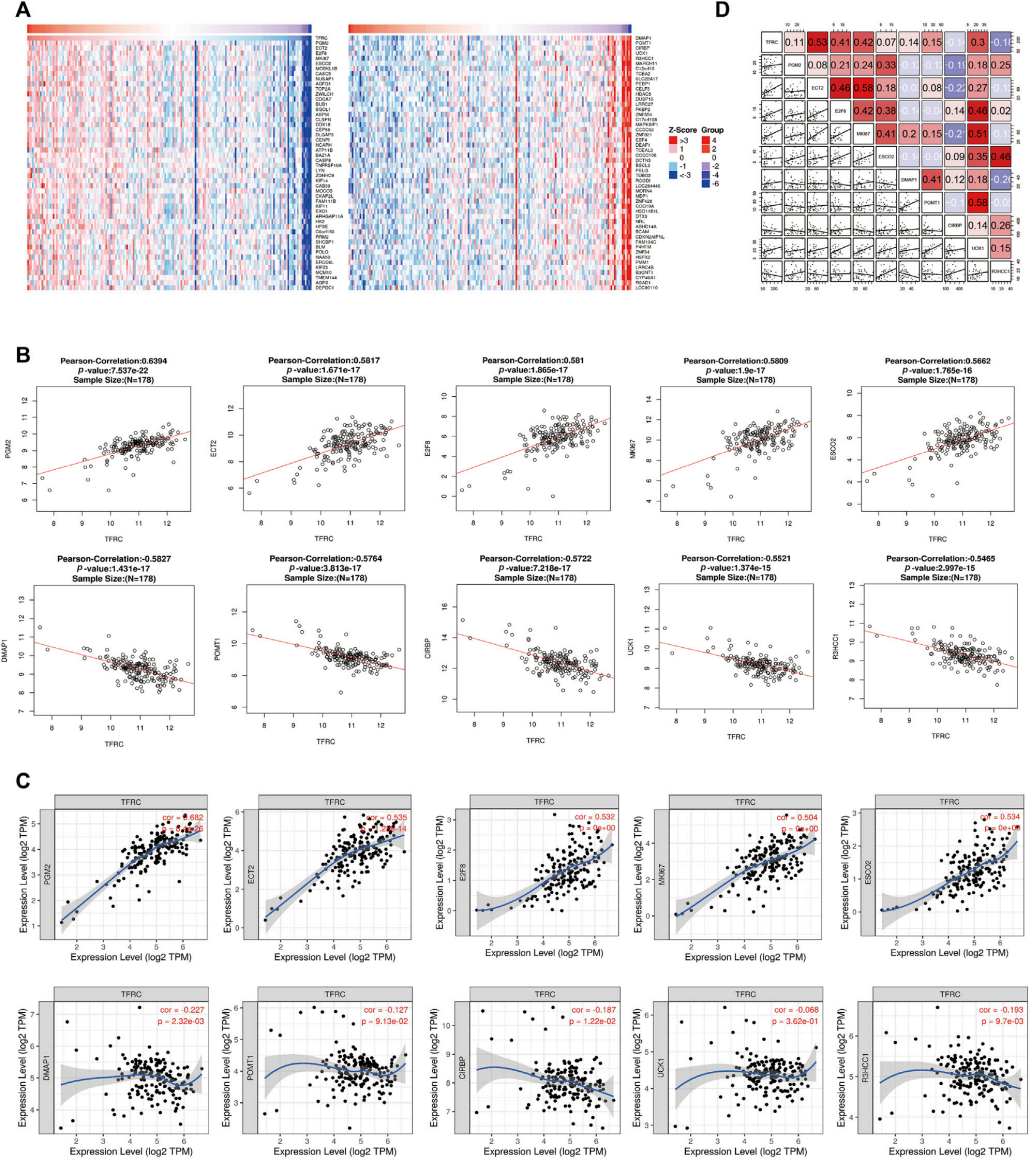
Correlative genes of TFRC in PAAD. **(A)** Heat map analysis of gene correlated with TFRC in pancreatic cancer samples. **(B)** The genes correlative with TFRC in PAAD (absolute Pearson’s r ≥ 0.5465) were assessed with the linkedomics database. TFRC was significantly correlated with PGM2 (r = 0.6392, *p* = 7.537e–22),ECT2 (r = 0.5817, *p* = 1.671e-17), E2F8 (r = 0.581, *p* = 1.865e-17), MK167 (r = 0.5809, *p* = 1.9e–17), ESCO2 (r = 0.5662, *p* = 1.765e–16), DMAP1 (r = −0.5827, *p* = 1.431e–17), POMT1 (r = −0.5764, *p* = 3.813e-17), CIRBP (r = −0.5722, *p* = 7.218e-17), UCK1 (r = −0.5521, *p* = 1.374e–15) and R3HCC1(r = −0.5465, *p* = 2.997e–15). **(C)** TFRC was significantly correlated with PGM2, ECT2, E2F8, MK167, ESCO2, DMAP1, POMT1, CIRBP, UCK1 and R3HCC1 in PAAD (via analysis in the TIMER database). **(D)** The relationship about TFRC and PGM2, ECT2, E2F8, MK167, ESCO2, DMAP1, POMT1, CIRBP, UCK1, R3HCC1 in 46 pancreatic cell lines.

To further investigate the possible effect of TFRC in PAAD, the genes that were co-expressed with TFRC were identified using the STRING website with TCGA data (Figure 3A). The top ten significant genes correlated with TFRC were selected (Figure 3B). The results indicated that TFRC was significantly correlated with PGM2 (r = 0.6392, *p* = 7.537e−22), ECT2 (r = 0.5817, *p* = 1.671e-17), E2F8 (r = 0.581, *p* = 1.865e-17), MK167 (r = 0.5809, *p* = 1.9e−17), ESCO2 (r = 0.5662, *p* = 1.765e−16), DMAP1 (r = -0.5827, *p* = 1.431e−17), POMT1 (r = -0.5764, *p* = 3.813e-17), CIRBP (r = -0.5722, *p* = 7.218e-17), UCK1 (r = -0.5521, *p* = 1.374e−15) and R3HCC1 (r = -0.5465, *p* = 2.997e−15). Furthermore, the correlation between *TFRC* and these genes was verified using TIMER (Figure 3C). Besides, we used the database to analyze TFRC and these 10 genes in 46 pancreatic cell lines. (Figure 3D). Based on the above results, we found that TFRC was strongly correlated with ECT2 and E2F8.

### Analysis of miRNA Co-Expressed With TFRC in PAAD

**FIGURE 4:**
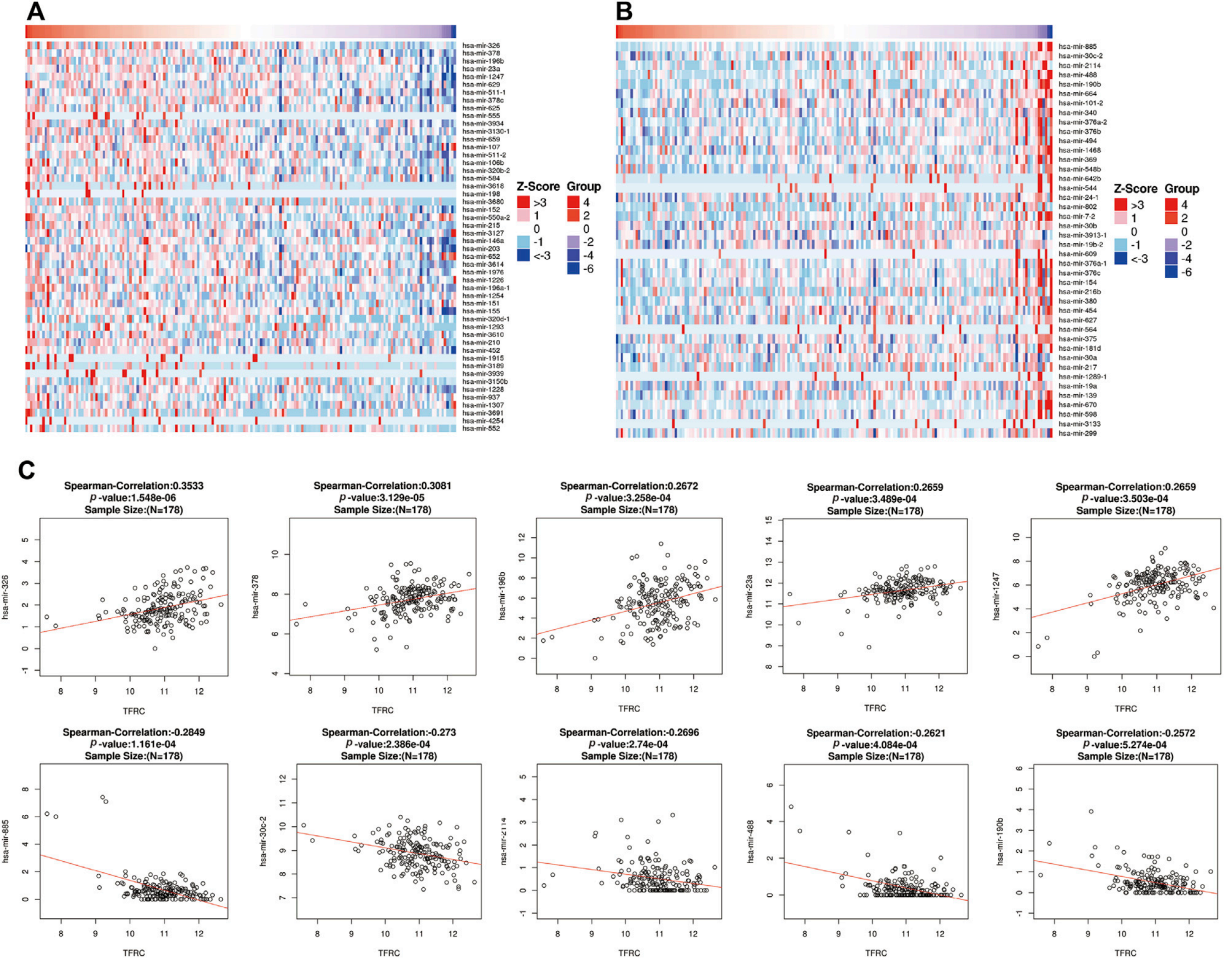
Correlative miRNAs of TFRC in PAAD. **(A,B)** Heat map analysis of miRNAs positively **(A)** and negatively **(B)** correlated with TFRC in pancreatic cancer samples. **(C)** The miRNAs correlative with TFRC in PAAD (absolute Pearson’s r ≥ 0.2391) were assessed with the STRING website. TFRC mainly interacts with mir-326, mir-378, mir-196, mir-23a, mir-1247, mir-885, mir-30c-2, mir-2114, mir-488 and mir-190b.

Also, in order to analyze the relationship between TFRC and related miRNAs in PAAD and explore the molecular mechanism of TFRC, the STRING website analysis found that TFRC mainly interacts with mir-326, mir-378, mir-196b, mir-23a, mir-1247, mir-885, mir-30c-2, mir-2114, mir-488, and mir-190b (Figures 4A–C).

### Protein Interaction Network Analysis of TFRC

**FIGURE 5:**
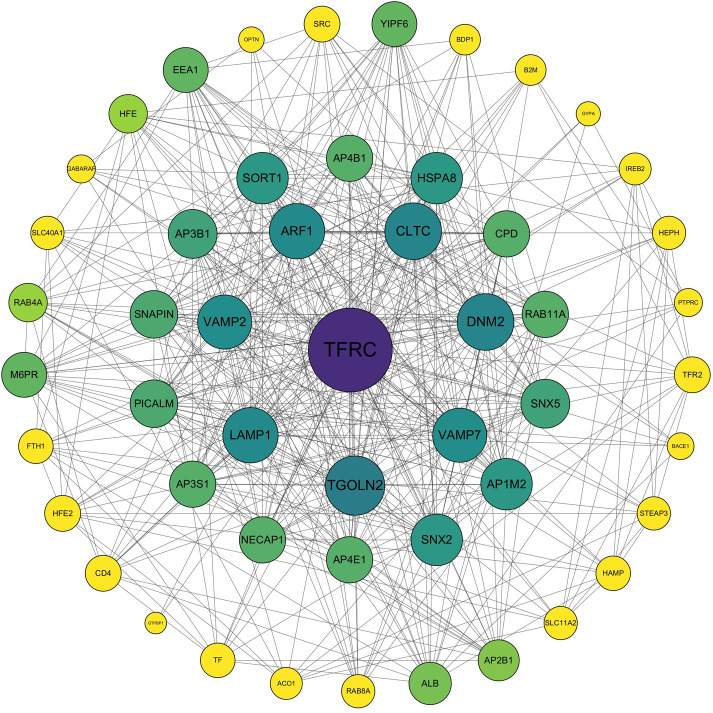
Protein-protein interaction network diagram of TFRC and related proteins.

To analyze the relationship between TFRC and related proteins and to further study the molecular mechanism of TFRC, STRING analysis was conducted. It was found that TFRC mainly interacts with proteins such as ARF1, TGOLN2, LAMP1, VAMP2, and DNM2 to exert its function (as shown in Figure 5).

### Enrichment Analysis of TFRC-Related Signaling Pathways

**FIGURE 6:**
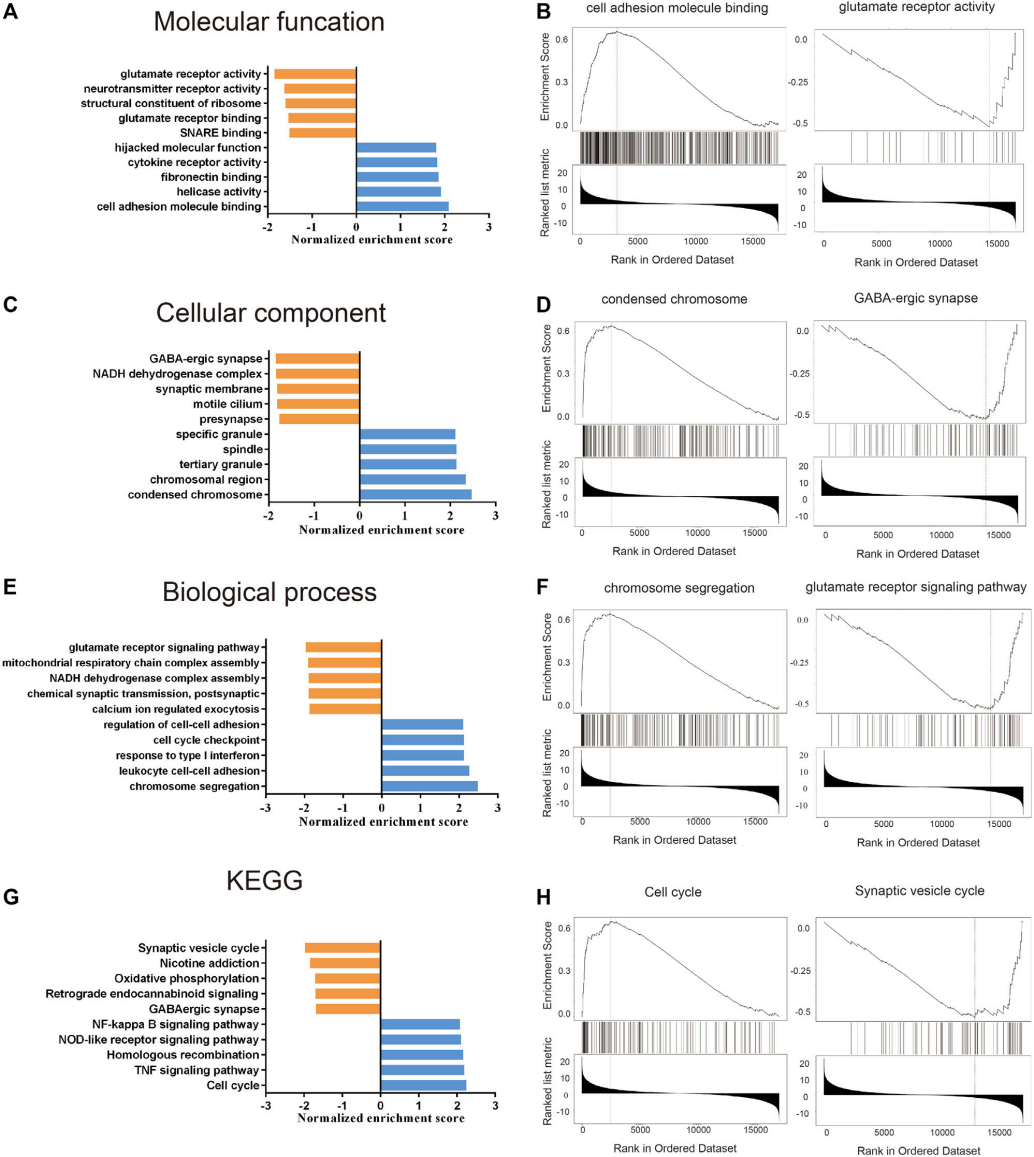
GO and KEGG enrichment analysis about TFRC based on the TCGA database. **(A–F)** GO enrichment analysis (including molecular function, cell components and Biological processes) about TFRC in PAAD. **(G,H)** KEGG enrichment analysis about TFRC in PAAD.

For better understanding of the molecular mechanism of TFRC, we performed GO (Table 2) and KEGG (Table 3; Figures 6G,H) enrichment analysis using the STRING website. In particular, the GO enrichment analysis divided the functional annotations of target genes into three categories: MF (Figures 6A,B), CC (Figures 6C,D), and BP (Figures 6E,F). In the KEGG and GO pathway analysis, some signaling pathways affected by TFRC are enriched during tumorigenesis. Cell adhesion molecule binding reflects the function of its surface position. Moreover, TFRC is associated with condensed chromosomes, chromosome segregation, and the cell cycle, so it may participate in cell division.

**TABLE 2:** GO analysis of TFRC and related genes.

ID	Description	Enrichment	*p*-Value	FDR
Molecular function							
GO:0050839	cell adhesion molecule binding	2.096207751	0	0
GO:0004386	Helicase activity	1.91677542	0	0.005593
GO:0001968	fibronectin binding	1.860041265	0	0.014915
GO:0004896	cytokine receptor activity	1.83212726	0	0.020276
GO:0104005	hijacked molecular function	1.813633957	0	0.02517
GO:0000149	SNARE binding	−1.50792551	0	0.262442
GO:0035254	glutamate receptor binding	−1.5356778	0.015444	0.247183
GO:0003735	structural constituent of ribosome	−1.605186136	0	0.169672
GO:0030594	neurotransmitter receptor activity	−1.624727485	0	0.209953
GO:0008066	glutamate receptor activity	−1.848385758	0	0.01831
Cellular component				
GO:0000793	condensed chromosome	2.46989535	0	0
GO:0098687	chromosomal region	2.34616898	0	0
GO:0070820	tertiary granule	2.138373842	0	0
GO:0005819	spindle	2.136836649	0	0
GO:0042581	specific granule	2.119246809	0	0
GO:0098793	presynapse	−1.7705986	0	0.006698
GO:0031514	motile cilium	−1.811511441	0	0.004386
GO:0097060	synaptic membrane	−1.812935078	0	0.005316
GO:0030964	NADH dehydrogenase complex	−1.837197398	0	0.005582
GO:0098982	GABA-ergic synapse	−1.839388382	0	0.009569
Biological process				
GO:0007059	chromosome segregation	2.486837573	0	0
GO:0007159	leukocyte cell-cell adhesion	2.267021237	0	0
GO:0034340	response to type I interferon	2.12437708	0	0
GO:0000075	cell cycle checkpoint	2.12356312	0	0
GO:0022407	regulation of cell-cell adhesion	2.108457936	0	0
GO:0017156	calcium ion regulated exocytosis	−1.868346869	0	0.003332
GO:0099565	chemical synaptic transmission, postsynaptic	−1.889148897	0	0.002314
GO:0010257	NADH dehydrogenase complex assembly	−1.891547023	0	0.003085
GO:0033108	mitochondrial respiratory chain complex assembly	−1.903830311	0	0.004165
GO:0007215	glutamate receptor signaling pathway	−1.965563938	0	0.001851

**TABLE 3:** KEGG pathway analysis of TFRC and related genes.

ID	Description	Enrichment	*p*-Value	FDR
hsa04110	Cell cycle	2.250961111	0	0
hsa04668	TNF signaling pathway	2.186933575	0	0
hsa03440	Homologous recombination	2.161490553	0	0
hsa04621	NOD-like receptor signaling pathway	2.10763845	0	0
hsa04064	NF-kappa B signaling pathway	2.077746125	0	0
hsa04727	GABAergic synapse	−1.687139775	0	0.031311
hsa04723	Retrograde endocannabinoid signaling	−1.705159004	0	0.030677
hsa00190	Oxidative phosphorylation	−1.715323871	0	0.036671
hsa05033	Nicotine addiction	−1.847367263	0	0.006876
hsa04721	Synaptic vesicle cycle	−1.971424675	0	0

### TFRC Predicts Immune Phenotypes in Pancreatic Cancer

**FIGURE 7:**
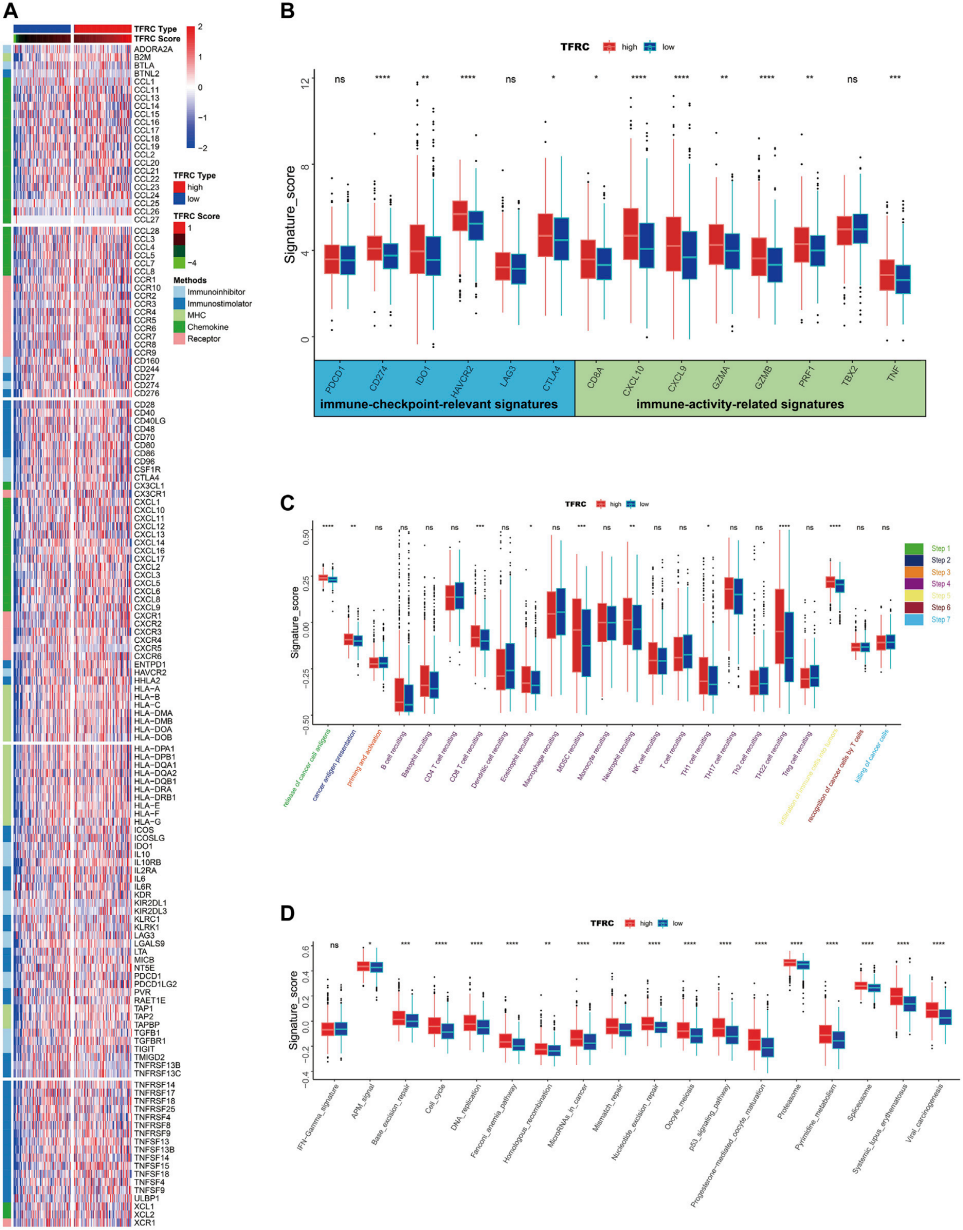
TFRC predicts immune phenotypes in pancreatic cancer. **(A)** Different expression of 144 immunomodulators (immune inhibitors, immune stimulators, MHC, chemokines and receptors) between high- and low-TFRC groups in pancreatic cancer. **(B)** Immune-checkpoint-relevant genes and immune-activation-relevant genes expressed in high- and low-TFRC groups. **(C)** Differences between high- and low-TFRC groups in each step of the cancer immune cycle. **(D)** Differences in immunotherapeutic pathway enrichment scores between high- and low-TFRC groups.

**FIGURE 8:**
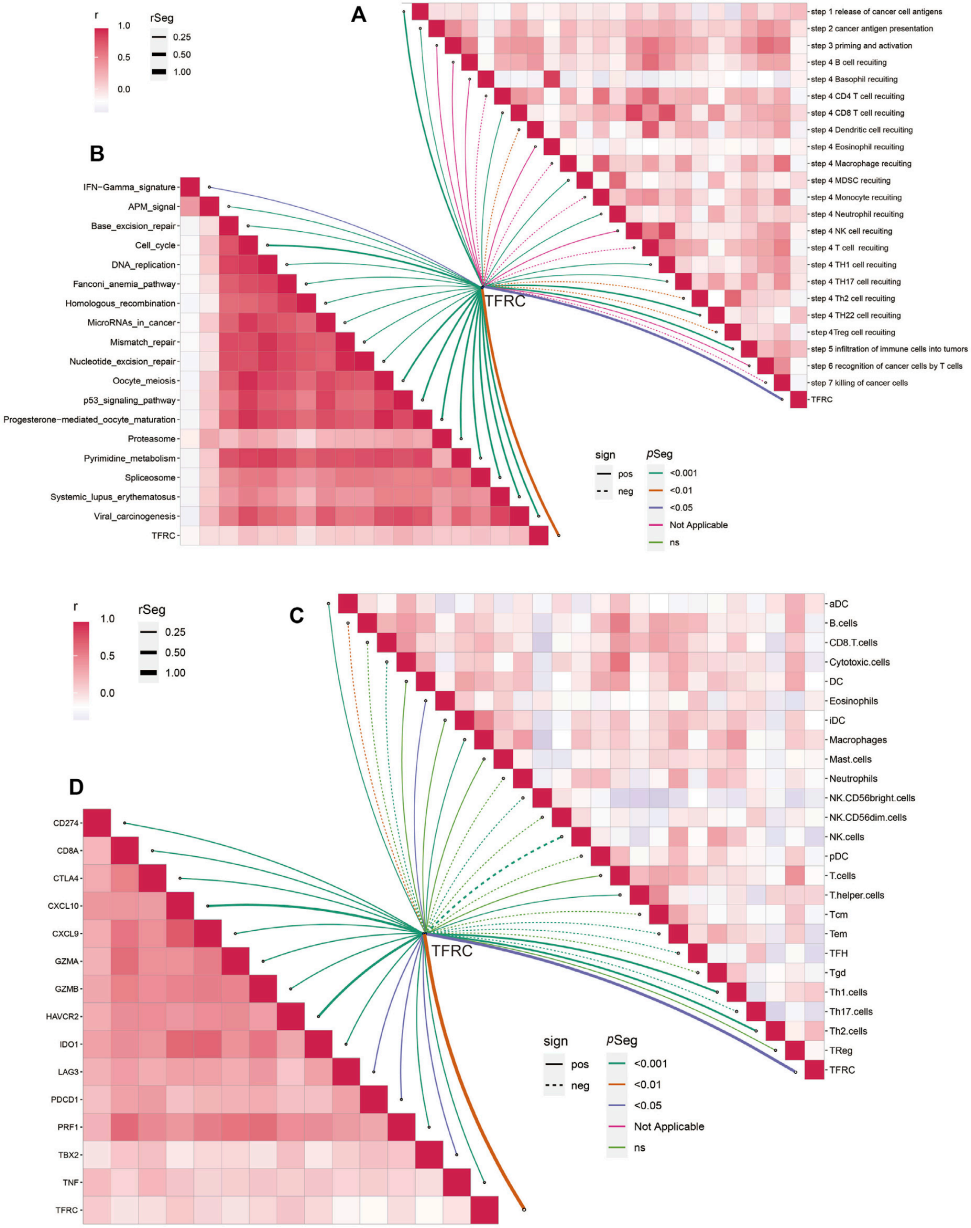
TFRC predicts immune phenotypes in pancreatic cancer. **(A)** Relationship between TFRC and various steps of the cancer immunity cycle. **(B)** Relationship between TFRC and the enrichment scores of immunotherapy-predicted pathways. **(C)** Relationship between TFRC and the tumor-infiltrating immune cell types. **(D)** Relationship between TFRC and Immune-checkpoint-relevant genes or immune-activation-relevant genes.

Firstly, we examined the relationship between TFRC and 144 immunomodulators, including immune inhibitors, immune stimulators, MHC, chemokines, and receptors in the TCGA cohort (Figure 7A). Then, we analyzed the immune activation of the high-TFRC and low-TFRC groups in the TCGA cohort. To this end, we selected several immune-related signatures, including immune-checkpoint-relevant signatures (CD274, CTLA4, HAVCR2, IDO1, LAG3, and PDCD1) and various immune-activity-related signatures (CD8A, CXCL10, CXCL9, GZMA, GZMB, PRF1, TBX2, and TNF). We used the Wilcoxon test to verify that the high-TFRC group had higher immune-related signatures, except for PDCD1, LAG3, and TBX2 (Figure 7B). Besides, we analyzed the activities of the cancer immunity cycles, which can evaluate the chemokine system and other immunomodulators. The high-TFRC group can activate several steps in the cycle, including the release of cancer cell antigens (Step 1), cancer antigen presentation (Step 2), trafficking of immune cells to tumors (Step 4) (CD8 T cells, MDSCs, neutrophils, TH1 cells, and TH22 cell recruiting), and infiltration of immune cells into tumors (Step 5) (Figure 7C). These steps may increase the number of infiltrating immune cells in the tumor microenvironment. Next, TFRC also enhances immunotherapy-positive gene signatures, including cell cycle, DNA replication, mismatch repair, P53 signaling pathway, and proteasome (Figure 7D). Subsequently, we used a butterfly heat map to determine the relationship between TFRC and the activities of the cancer immunity cycle (Figure 8A), immunotherapy-positive gene signatures (Figure 8B), immune cell infiltration (Figure 8C), and immune-relevant genes (Figure 8D) in PAAD. Overall, TFRC can used to predict immune phenotypes, molecular subtypes in PAAD, which can lay a foundation for new immunotherapies for pancreatic cancer.

### TFRC Evaluates Immune Cell Infiltration and Immunotherapy

**FIGURE 9:**
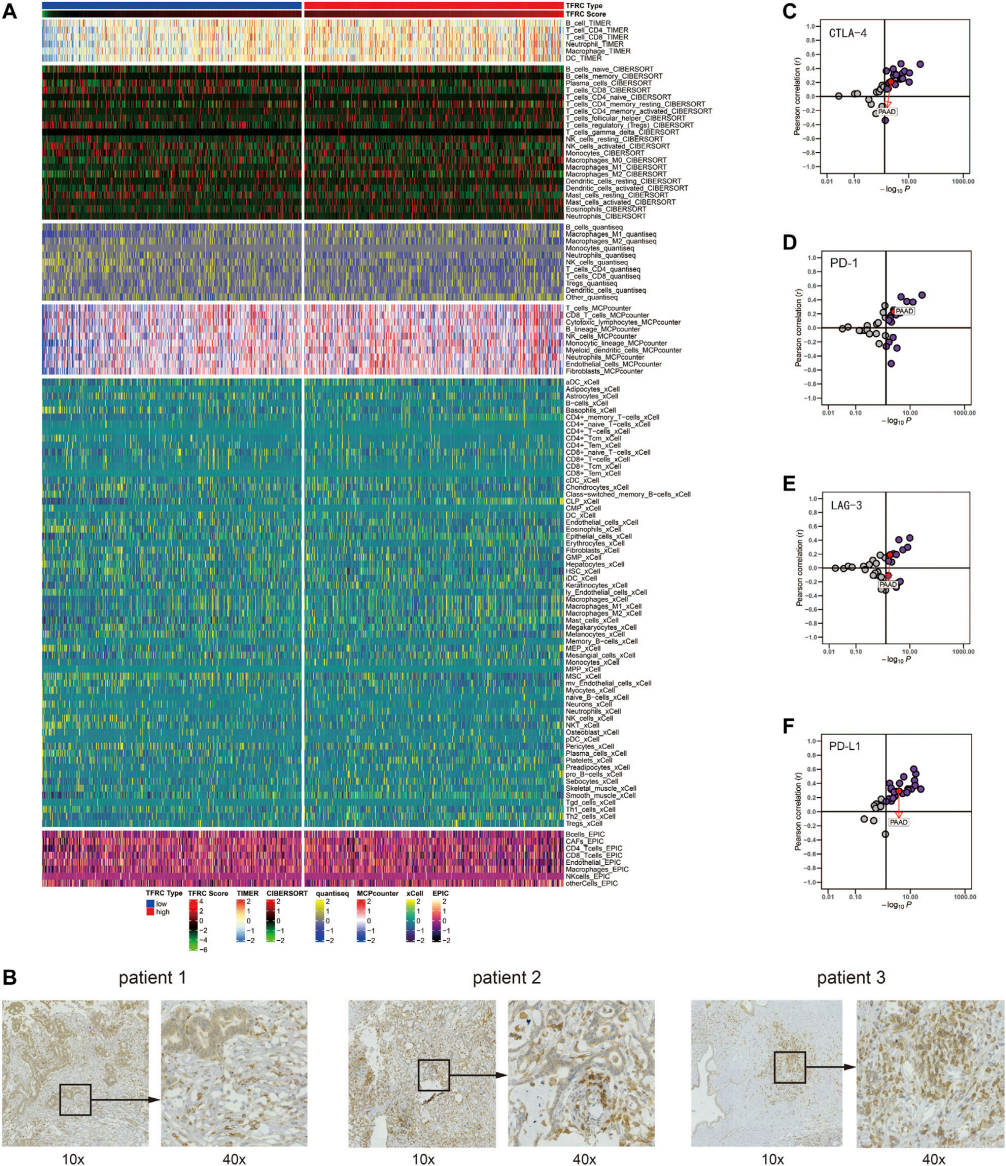
TFRC predicts immune cells infiltration in pancreatic cancer. **(A)** Correlation between TFRC and the infiltrating immune cells with six algorithms: TIMER, CIBERSORT, quantiseq, MCPcounter, xCell, and EPIC. **(B)**. Representative IHC staining of CD3 in pancreatic cancer tissue. **(C–F)** Relationship between TFRC and CTLA-4, PD-1, LAG-3, PD-L1 in pan-cancer.

**FIGURE 10:**
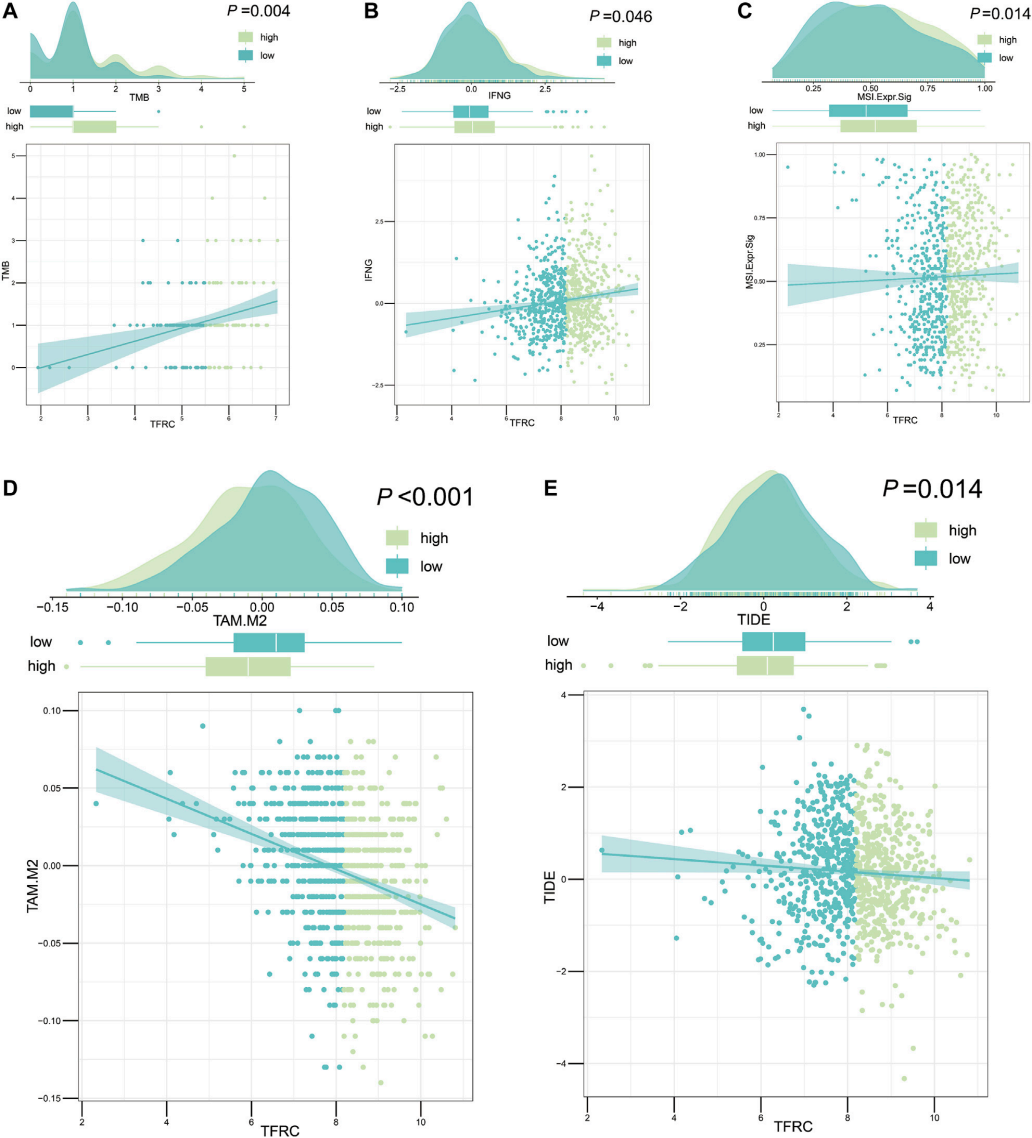
TFRC evaluates immunotherapy about pancreatic cancer. **(A–C)** TFRC was positively correlated with tumor mutation burden (*p* = 0.004) **(A)** interferon-gamma signature (*p* = 0.046) **(B)** and microsatellite instability signature (*p* = 0.014) **(C)**. **(D,E)** TFRC was negatively correlated with tumor associated macrophage M2 type (*p* < 0.001) **(D)** and TIDE score (*p* = 0.014) **(E)**.

After analyzing the correlation between TFRC and immunophenotype, to further explore the relationship between TFRC and immunotherapy, we firstly calculated the immune cells infiltrating the tumor microenvironment on the strength of bulk RNA-seq data by using several algorithms (TIMER, CIBERSORT, quantiseq, MCPcounter, xCell, and EPIC) (Figure 9A). As a result, we found that the high expression levels of TFRC means more infiltrating immune cells. To verify the results of data analysis, we selected a common immune cell marker CD3 for immunohistochemical tests on tissues from three pancreatic cancer patients with high TFRC expression and observed numerous inflammatory infiltrates (Figure 9B). For the current common immunotherapy methods, we also demonstrated that TFRC was co-expressed with various immune checkpoints in several cancers, including PAAD (Figures 9C–F). Besides, tumor mutation burden (TMB) has always been considered as one of the criteria for immunotherapy, so we analyzed the TMB and found that it was positively correlated with TFRC (Figure 10A). Interferon-gamma (IFNG) signature and microsatellite instability (MSI) signature are also used to predicted the immunotherapy (Fu et al., 2020), so we used them to predict and got the same conclusion (Figures 10B,C). In addition, Peng Jiang (Jiang et al., 2018)developed TIDE (a computational method to model two primary mechanisms of tumor immune evasion), we found tumor associated macrophage M2 type (Fu et al., 2020) and TIDE score were negatively correlated with TFRC (Figures 10D,E), which further demonstrates the relationship between TFRC and immunotherapy.

## Discussion

Pancreatic cancer is a malignant tumor of the digestive tract and has a high mortality rate (Ansari et al., 2016; Mizrahi et al., 2020). China is a country with a high incidence of pancreatic cancer, which causes a serious social burden due to its high morbidity, mortality, and poor prognosis (Ilic and Ilic, 2016). In recent years, the treatment and prognosis of pancreatic cancer patients have improved given the improvements in treatment methods, but the survival rate is still low (Chu et al., 2017; Pereira et al., 2020; Wolfgang et al., 2013). Therefore, it is crucial to identify neutral biomarkers and related treatments for pancreatic cancer. In this study, we used bioinformatics and biochemical experiments to verify that the expression level of TFRC was increased in pancreatic cancer tissues and was associated with a poor prognosis. In addition, TFRC expression was significantly associated with clinical characteristics. We also analyzed the GO and KEGG pathways of TFRC and constructed a protein interaction network for TFRC. Cell adhesion molecule binding, helicase activity, fibronectin binding, cytokine receptor activity, chromosome segregation, leukocyte cell-cell adhesion, response to type I interferon, cell cycle checkpoint, and regulation of cell-cell adhesion may be related to signaling pathways regulated by TFRC in pancreatic cancer. These results indicate that TFRC participates in the formation of pancreatic cancer, which may be used as a biomarker and therapeutic site for a pancreatic cancer. Moreover, TFRC can be used for prediction of immune phenotypes and immune cell infiltration in pancreatic cancer. TFRC is positively associated with many immunomodulators and is co-expressed with several significant immune checkpoints. The cancer immunity cycle can also be activated in high-TFRC stations, and most immunotherapy-positive genes are high. Overall, TFRC can be applied to predict immune phenotypes, molecular subtypes, and immune cell infiltration in PAAD, which, in turn, can create a basis for new immunotherapies for pancreatic cancer in the future.

**FIGURE 11:**
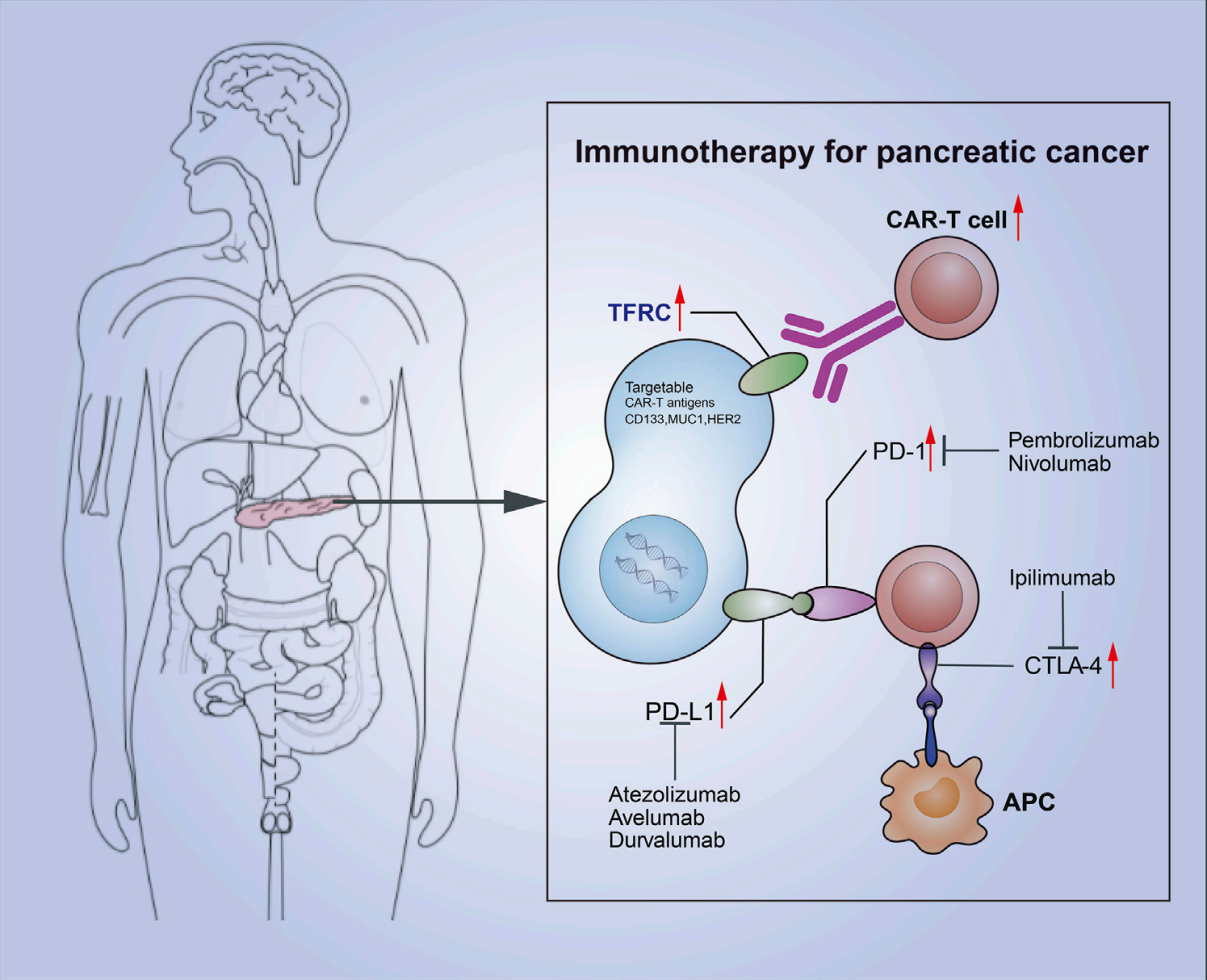
Combination of TFRC-associated CAR-T and immune checkpoint suppression for pancreatic cancer.

Some previous studies have classified cancers according to their immune infiltration (Nuti et al., 2018). The system, which classifies cancers through immunity rather than the cancer-based tumor classification, effectively introduces the concepts of “hot” (highly invasive) and “cold” (non-invasive) tumors (Galon and Bruni, 2019; Nuti et al., 2018). Pancreatic cancer is a highly malignant cancer with less immune invasion and is often classified as a cold tumor. At present, a combination of multiple immunotherapies is often used to overcome the lack of immune response in cold tumors and transform cold tumors into hot tumors. In pancreatic cancer, TFRC is associated with the tumor immune cycle by analyzing the relationship between TFRC and the immune cycle. The high-TFRC group recruited more immune cells, and infiltrating immune cells (dendritic cells, macrophages, Th1 cells, and Th2 cells) was increased in the high-TFRC group. Therefore, high TFRC expression often leads to a relatively “hot” tumor microenvironment. The relative efficacy of immunotherapy may be better due to the presence of a relatively high number of infiltrating immune cells and increased immune checkpoints. Moreover, TFRC, as a molecular protein on the cell surface that includes cell adhesion molecule binding, may be used in the future for CAR-T therapy related to pancreatic cancer immunotherapy (by targeting TFRC to induce the patient’s own immune cells to attack the tumor). We can apply these two immunotherapies together by using this method to unravel new therapeutic possibilities for patients with pancreatic cancer (Figure 11). Thus far, our laboratory has carried out CAR-T therapy, while the combination therapy would be clinically validated in the future.

TFRC has been also extensively studied in other cancers. In the nervous system, the N-Myc proto-oncogene (MYCN) maintains TFRC expression in proliferating neural cells (Menon et al., 2019). Additionally, a subtype of TFRC is positively associated with the infiltration abundances of immune cells in breast cancer, and it is also considered to be a potential target for immunotherapy in breast cancer (Chen et al., 2021) (Corte-Rodríguez et al., 2019). Some studies have discovered that TFRC controls malignant behavior and stemness of tumor stem cells in hepatocellular carcinoma by regulating iron accumulation, which may improve therapeutic approaches (Xiao et al., 2020). In human fibrosarcoma cells, TFRC is a specific ferroptosis marker (Feng et al., 2020). TFRC is also highly expressed in hematological tumors; erythroid lineage cells and proliferating cells both have high surface TFRC expression. TFRC is the highest in T lymphoblasts (Acharya and Kala, 2019). Therefore, the findings of our current study should be comprehended alongside with the previous studies’ results, as we found that TFRC is involved in the occurrence and development of many cancers. In future studies, it is very important to focus on the basic and clinical research of TFRC.

The drawback of this study stems from the fact that the relevant data were obtained only from online databases. Although the results have certain suggestive significance, they also have certain limitations and need to be combined with relevant *in vitro* and *in vivo* experiments and practical results of the clinical studies should be further verified. This study can become an important step for the following basic and clinical studies of TFRC in pancreatic cancer, thus, further providing scientific arguments and evidences. Further studies are needed to explore the function of TFRC in pancreatic cancer as they will help to generate new ideas for the early diagnosis, targeted therapy, and prognosis judgment of patients.

## Conclusion

To conclude, we used bioinformatics and biochemical experiments to verify that the level of TFRC is higher in pancreatic cancer than that in healthy pancreas. High-TFRC is also associated with a poor prognosis and is related to tumor immunology, suggesting that TFRC may be a potential prognostic molecular predictor for pancreatic cancer patients.

## Data Availability

The datasets presented in this study can be found in online repositories. The names of the repository/repositories and accession number(s) can be found below: https://www.ncbi.nlm.nih.gov/genbank/, 7037.
